# Do I really feel it? The contributions of subjective fluency and compatibility in low-level effects on aesthetic appreciation

**DOI:** 10.3389/fnhum.2015.00373

**Published:** 2015-06-26

**Authors:** Michael Forster, Wolfgang Fabi, Helmut Leder

**Affiliations:** Department of Basic Psychological Research and Research Methods, Faculty of Psychology, University of ViennaVienna, Austria

**Keywords:** ease of processing, feeling of fluency, aesthetic appreciation, liking, compatibility

## Abstract

The causes for the liking of objects are multifaceted. According to the processing fluency account, the ease with which an object is processed leads to a subjective feeling of fluency. This subjective feeling is then interpreted as a positive reaction toward the object resulting higher liking. However, evidence regarding the processes underlying this relation is scarce. To show that the subjective feeling can indeed be responsible for liking, we experimentally manipulated processing ease by providing false physiological feedback (varying skin conductance indicated varying feelings of fluency) and by varying presentation times between 100 and 400 ms while participants viewed line drawings of objects and rated them for liking. A first experiment showed that both false physiological feedback and presentation duration influenced liking. Stimuli primed with a (fake) visualization of a physiological correlate of high ease of processing were liked more than stimuli primed with a low ease of processing. Liking ratings in a no-feedback condition fell between the high and low feedback conditions. To explore possible compatibility effects of coupling visual feedback to the fluency interpretation, in a second experiment we reversed the feedback interpretation—visualization of high skin conductance now indicated low ease of processing. The results show a similar pattern, though the effect was subtler. This indicates that when the coupling of feedback to fluency is less apparent or less compatible, the feeling is less strongly linked to liking. Our results support the claim that variations in the feeling of fluency affect the appreciation of objects in terms of liking. Together, the experiments suggest the contributions of processing ease as well as compatibility to the experience of liking.

## Introduction

Current models of aesthetic appreciation indicate that liking is the result of multiple processing stages operating at higher, but also at lower, cognitive levels (Leder et al., 2004; Jacobsen, 2006; Pelowski and Akiba, 2011). For the effect of low-level properties, such as contrast (Reber et al., 1998) or symmetry (Bertamini et al., 2013; Gartus and Leder, 2013), on liking the theory of processing fluency provides a general explanatory framework (for reviews see Reber et al., 2004a; Alter and Oppenheimer, 2009). The theory states that the ease with which an object (an artwork, a product, or any other scenery) is processed is accompanied by a subjective feeling of fluency, which serves as a basis for several kinds of judgments and evaluations (Reber et al., 2004b; Forster et al., 2013; Garcia-Marques et al., 2013). This subjective feeling of fluency denotes the experience of variations in the ease of processing. As there are no objective feelings of fluency, the addition of *subjective* strictly speaking is obsolete. It is, however, commonly used to highlight the *experience* of the subject. Therefore, in the following we call this experience the subjective feeling of fluency, or in short, felt fluency.

In a typical experiment on processing fluency, the presentation of images, words, or sentences is manipulated to create conditions of lesser and greater ease of processing. Variations in presentation duration (Forster et al., 2013), contrast (Reber et al., 1998), or clarity (Reber et al., 2004b) are commonly used. Though the processing fluency theory is not limited to a specific level or sensory domain of processing, experiments have mainly tested the impact of the ease of visual processing (i.e., perception) or the ease of memory retrieval (i.e., remembering or recognition). In this article we focus on the ease of visual processing as well.

Participants are able to consciously report variations in felt fluency coming along with variations in processing ease. Furthermore, higher ease of processing, in turn, influences various evaluations, such as liking (Reber et al., 1998; Forster et al., 2013), truth (Dechêne et al., 2010), familiarity (Whittlesea, 1993), or even sensory stimulus properties such as brightness (Mandler et al., 1987) or loudness (Jacoby et al., 1988). Often, the two outcomes of a manipulation of ease of processing, felt fluency and liking, are also positively correlated with each other (see for example Forster et al., 2013). It is, however, still unclear whether we really use the feeling of fluency, i.e., the conscious representation of the ease of processing, for our evaluations. We thus intend to test whether the feeling of fluency indeed influences the liking of a stimulus. In two experiments, we tested whether subjectively felt fluency is indeed a source for our evaluations. Consequently, we contribute to the claim that even such “cold” cognitive processes as perception—or more specifically the dynamics of perception—can influence our aesthetic appreciation of objects (Greifeneder et al., 2011).

According to the theory, the easier something is to process, the more it is liked (Reber et al., 2004a). This is because the feeling of fluency is claimed to be *per se* positive, because it signals a positive state of affairs, error free processing, and absence of threat (Winkielman et al., 2003). This positivity is then attributed to a positive reaction toward the object (Winkielman and Cacioppo, 2001; Topolinski et al., 2009). Others claim that the feeling of fluency is an affectively unspecific activation attributed to a higher evaluation of the object (Bornstein and D'Agostino, 1992, 1994) or that fluency amplifies affective evaluations—positive stimuli are regarded as more positive, negative stimuli as more negative (Albrecht and Carbon, 2014). Conclusive evidence is still lacking. A necessary first step, however, is to understand whether and how the subjective feeling of fluency exerts its influence on people's evaluations.

Most researchers agree that there is a feeling of fluency, i.e., a subjectively experienced representation of the ease of processing (Reber et al., 2004b; Regenberg et al., 2012; Forster et al., 2013; Jakesch et al., 2013). Though the subjective feeling of fluency is not a necessary precondition for effects of ease of processing on liking—ease of processing not necessarily needs to become conscious (Reber et al., 2002; Topolinski and Strack, 2009)—the feeling of fluency was found to be correlated with liking and usually can become conscious (Reber et al., 2004b; Forster et al., 2013; Jakesch et al., 2013). Although Bornstein and D'Agostino (1992, 1994) argued that conscious reflection on ease of processing leads to a discounting of the ease, and thus eliminates effect on the evaluations, others have failed to show that discounting is an issue (Newell and Shanks, 2007; Forster et al., 2013). Indeed, felt fluency seems to have an even tighter connection to liking than an actual manipulation of ease of processing (Forster et al., 2013). We thus tested whether making participants explicitly believe their feeling of fluency is affected does influence their evaluations of felt fluency and liking.

In two experiments we gave participants false feedback about their ease of processing after viewing a stimulus (Valins, 1966). We told participants that their ease of processing could be objectively measured by recording their skin conductance response (SCR) to the stimuli. In Experiment 1, in each trial our participants received either a fake feedback of high processing ease (increasing SCR curve), a fake feedback of low processing ease (decreasing SCR curve), or, as a control, no feedback.

For the false feedback manipulation to work properly, it was paramount that our participants believed the feedback. Thus, in post-questions we tested whether the participants were naïve to the purpose of the experiment. Prior to data analysis, we excluded all participants who explicitly indicated that they did not believe the instruction or that they explicitly ignored the feedback. The main results are thus only from participants who believed the instruction. For the sake of complete reporting, the analysis including all participants can be found in a Supplementary Material.

A finding that feedback of an increasing SCR leads to an increase in liking, and vice versa, could also be caused by a mere compatibility between the SCR curve and the response (Slovic et al., 1990). To exclude such an explanation, in a second experiment we reversed the meaning of the SCR feedback: a decrease in the SCR curve now indicated a higher ease of processing, which should lead to higher liking, and vice versa. We thus switched the mapping between the SCR feedback and the participants' response. In both experiments, the crucial comparison is between the high and low feedback conditions. If in both experiments a high feedback compared to a low feedback leads to higher felt fluency and liking ratings then the conscious reflection of the subjective feeling of fluency does influence liking.

As a second source of the subjective feeling of fluency in both experiments we manipulated the actual ease of processing by varying the presentation duration of the images between 100 and 400 ms, a manipulation proven successful in previous studies on fluency (Reber et al., 1998; Winkielman and Cacioppo, 2001; Forster et al., 2013). The longer the stimuli are presented, the more information can be gathered, the easier they are to process (Mackworth, 1963), and the higher the felt fluency. Thus the felt fluency participants could use as a source for their evaluations consisted of the feeling derived from an actual variation in ease of processing and of the false feedback. An effect of the variations in presentation duration on liking should replicate earlier findings that longer presentation durations and thus higher ease of processing is linked to liking (Reber et al., 1998; Winkielman and Cacioppo, 2001; Forster et al., 2013).

Having two sources of felt fluency—presentation duration and feedback—furthermore allows studying how these two sources interact. If we find an effect of feedback alone, it would mean that a conscious representation of felt fluency overrides the actual presentation duration manipulation of ease of processing and governs the liking evaluation. If both the feedback and the presentation duration have an effect or if they interact—feedback of high ease of processing amplifies the effects of presentation duration on liking and feedback of low ease of processing attenuates the effects of presentation duration—then both sources of felt fluency have their share in the evaluations. If the false feedback leaves liking uninfluenced, then the conscious representation leads to discounting of feelings of fluency as a source for our evaluations (Bornstein and D'Agostino, 1992, 1994).

## Experiment 1: Materials and methods

### Participants

In Experiment 1, 58 participants believed the feedback manipulation and were thus eligible for further analysis. The sample consisted of volunteers recruited by the experimenter or undergraduate students from the University of Vienna participating in return for partial course credit. Three participants were removed from the final sample, because they failed to show sufficient visual acuity (tested before the experiment). Thus, the final sample consisted of 55 participants (46 female, *M*_age_ = 23.4 years, *SD* = 4.5). All participants provided written informed consent prior to their participation. Participants were informed that participation and data collection were fully anonymous and that they could withdraw at any time during the experiment without any further consequences. The experiments were conducted in accordance with the Declaration of Helsinki (revised, 1983) and the guidelines of the Faculty of Psychology, University of Vienna. According to the Austrian Universities Act 2002 (UG2002), which was active at the time the experiments were performed, only medical universities were required to appoint ethics committees for clinical testing, application of medical methods and applied medical research. Therefore, no ethical approval was sought.

### Stimuli

**Figure 1:**
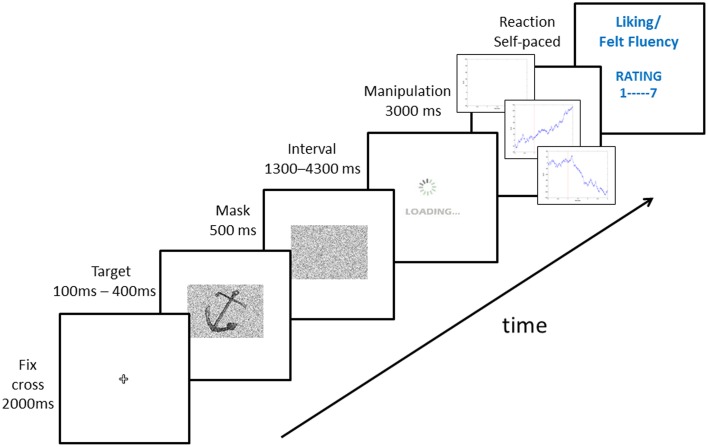
**Sequence of a trial in our experiments**.

For the experimental trials we used a selection of 120 line drawings of common objects (Rossion and Pourtois, 2004). As the original images were very easy to perceive, all images were overlaid with 60% Gaussian noise in Adobe Photoshop. This also improved the plausibility of our manipulation of the ease of processing. For the practice trials, 9 famous images (for example the Coca-Cola logo, the Mona Lisa) and 9 non-famous images were selected as targets. For the fake skin conductance response (SCR) feedback we generated 200 random wave patterns with Matlab 7.14 (code available upon request). The code was designed to generate images closely resembling a physiological response (see Figure 1 or **Figure 3**). Out of the 200 images we then chose 40 images, which best represented an SCR increase, and 40 images, which best represented an SCR decrease.

### Design and procedure

As a manipulation of the actual ease of processing, we varied the presentation duration of the stimuli among 100, 200, 300, and 400 ms (Forster et al., 2013). For each participant these presentation durations were randomly assigned to the stimuli with the constraint that each of the presentation durations in the end was presented equally often. As a feedback manipulation in each trial after the stimulus presentation, participants received either a feedback of an increasing skin conductance response, representing a high ease of processing feedback, a feedback of a decreasing skin conductance response, representing a low ease of processing feedback, or no feedback. The actual feedback curve image was randomly picked out of the set of possible increasing or decreasing curve images. The type of feedback was randomly assigned to the presentation durations with the constraint of ensuring that for each of the durations each type of feedback was presented equally often. Thus, the factors *presentation duration* and *feedback* were fully crossed.

Upon arrival our participants signed the consent form and performed standardized tests for visual acuity and color vision. Then participants were familiarized with the equipment and the tasks. For our fake physiological measures, we used an 8-channel bioamplifier (Mobi8-BP, TMSI B. V., Enschede, The Netherlands). First, two flat Ag/AgCl-electrodes were placed at the medial phalanges of digits III and IV of the non-dominant hand. As no recording setup was preloaded on the device, no data were recorded. Apart from this fact, everything else was identical to an actual measure of skin conductance. This aimed to increase the credibility of the manipulation.

Prior to the experiment, participants were also familiarized with the plots of the SCR response and participants were led to believe that previous studies have shown that high ease of processing goes along with an increased SCR response. To render the manipulation even more credible 18 practice trials were administered where 6 very famous images (as for example the Mona Lisa) were followed by an increasing SCR feedback and 6 non-famous images were followed by a decreasing SCR feedback. The rest of the images—3 famous and 3 non-famous—were followed by no feedback.

After these familiarization trials, the main experiment begun. In each trial, a fixation cross was first presented for 2000 ms followed by the target for either 100, 200, 300, or 400 ms. Then a mask consisting of random noise was presented for 500 ms to limit visual processing. Then an animated loading screen indicating that the SCR response is being generated was presented for a random time between 1300 and 4300 ms. Both the loading screen and the random time interval were included to make the computation of an actual SCR feedback more plausible. The loading screen was followed by the SCR feedback image presented for 3000 ms. Finally, participants first indicated how much they liked the target (on a 7-point scale ranging from 1 *not at all* to 7 *very much*) and then indicated their felt fluency, i.e., how easy the processing of the stimulus has been (also on a 7-point scale ranging from 1 *not at all* to 7 *very much*, see Figure 1 for an illustration of a trial sequence). The felt fluency measure also served as a manipulation check for the false feedback.

In total, participants performed 120 trials. In the end the participants were thanked for their participation and thoroughly debriefed about the purpose of the experiment. The whole experiment took around 30 min and was run with e-prime 2.0 (Schneider et al., 2002) on a display with a resolution of 1280 × 1024 pixels at 60 Hz.

## Experiment 1: Results and discussion

To investigate the effects of a false feedback and an actual variation in ease of processing, we performed two separate repeated measures analyses of variance (ANOVAs). First, as a manipulation check, we performed a 4 (presentation duration: 100, 200, 300, or 400 ms) × 3 (feedback: high, low, or absent) ANOVA with ratings of felt fluency as the dependent variable. To investigate whether our manipulations lead to effects in the judged liking of the stimuli the same ANOVA was run with liking as the dependent variable. For the ANOVA we first averaged the ratings of each participant over stimuli separately for the four presentation durations and the three feedback conditions. For the overall means sampled over participants and stimuli see Table 1. In all analyses, the level of statistical significance was *p* < 0.05. When the sphericity assumption was not met, the Greenhouse–Geisser correction was applied and the corrected degrees of freedom are reported. For analyses of presentation duration, we performed linear trend analyses, given the hypothesis that ratings of felt fluency and liking should linearly increase with longer presentation durations. For the analysis of the feedback, we compared the mean ratings in the low feedback condition with the mean ratings in the high feedback condition employing *t*-tests.

**Table 1:** **Means and standard deviations (in parentheses) for ratings of felt fluency and liking in both experiments separately for presentation duration and feedback**.

**Rating**		**Experiment 1**			**Experiment 2**		
		**Low EOP**	**No FB**	**High EOP**	**Low EOP**	**No FB**	**High EOP**
Felt fluency	100	4.32 (1.07)	4.51 (1.08)	4.80 (0.83)	4.38 (1.06)	4.51 (0.94)	4.69 (1.00)
	200	4.58 (0.93)	4.81 (0.81)	5.07 (0.90)	4.64 (0.94)	4.97 (0.82)	5.12 (0.92)
	300	4.70 (1.02)	4.92 (0.81)	5.15 (0.81)	4.97 (0.87)	5.07 (0.94)	5.27 (0.82)
	400	4.83 (1.10)	5.07 (0.85)	5.24 (0.80)	4.89 (0.98)	5.31 (0.87)	5.38 (0.93)
Liking	100	3.57 (0.94)	3.81 (0.98)	3.84 (0.97)	3.57 (1.04)	3.59 (0.90)	3.68 (0.97)
	200	3.72 (0.98)	3.78 (0.97)	3.86 (0.99)	3.69 (1.03)	3.81 (1.04)	3.73 (0.95)
	300	3.72 (0.87)	3.83 (0.95)	3.96 (1.06)	3.79 (0.96)	3.77 (0.99)	3.85 (1.02)
	400	3.85 (0.94)	4.00 (1.00)	4.01 (1.05)	3.74 (1.02)	3.91 (0.95)	3.93 (0.97)

*Low EOP, feedback curve indicating a low ease of processing; No FB, no-feedback curve; High EOP, feedback curve indicating a high ease of processing*.

### Felt fluency

**Figure 2:**
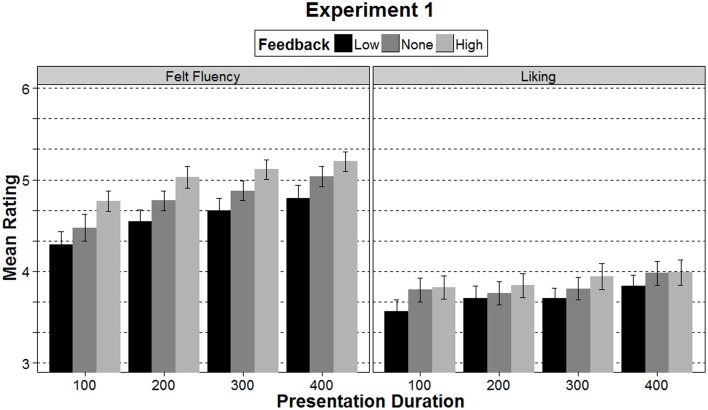
**Mean ratings of felt fluency and liking in Experiment 1 separately for the three feedback conditions and the four presentation durations**. Error bars represent ±1 SE.

The repeated measures ANOVA with felt fluency as the dependent variable showed significant main effects of both presentation duration, *F*_(2.4, 128.39)_ = 19.94, *p* < 0.001, η^2^_p_ = 0.27, and feedback, *F*_(1.30, 70.11)_ = 15.29, *p* < 0.001, η^2^_p_ = 0.22, but no interaction, *F*_(4.70, 253.54)_ = 0.17, *p* = 0.970, η^2^_p_ = 0.003. Linear trend analysis shows that ratings of felt fluency linearly increased with longer presentation durations, *F*_(1, 54)_ = 37.99, *p* < 0.001, η^2^_p_ = 0.41 (see Table 1 and Figure 2). For the feedback manipulation, a *t*-test showed that ratings of felt fluency were significantly higher in the high feedback condition (*M* = 5.07, SD = 0.72) than in the low feedback condition (*M* = 4.61, SD = 0.90, *p* < 0.001). Interestingly, the mean in the no-feedback condition lay between the two feedback conditions (*M* = 4.83, SD = 0.75; no vs. high, *p* < 0.001, no vs. low, *p* = 0.006, see Figure 2). In line with the manipulation intention, with longer presentation durations and with high SCR feedback stimuli were judged as easier to perceive than with shorter presentation durations and with low SCR feedback. The facts that ratings in the no-feedback conditions were numerically between high and low feedback and that high and low feedback significantly differed from each other indicate that we successfully made our participants believe that the feedback reflected their ease of processing.

### Liking

**Figure 3:**
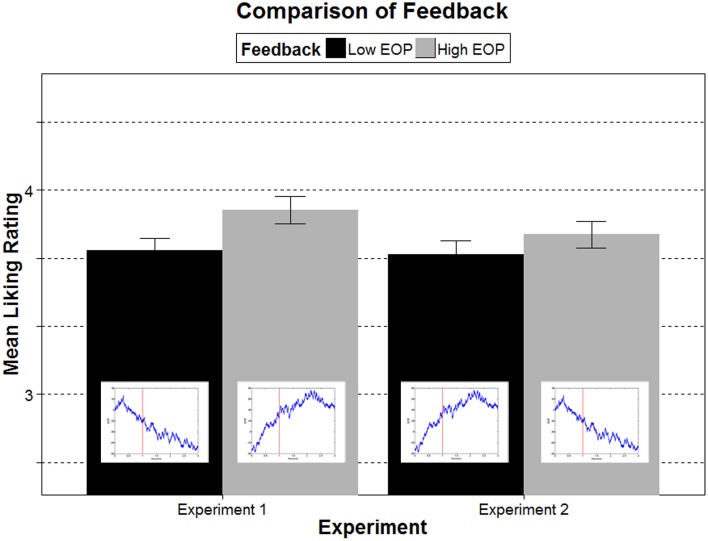
**The effects of false feedback show that in both experiments mean liking was higher following a feedback of high ease of processing compared to low ease of processing**. This effect did not depend on the mapping between the SCR curve and the ease of processing. Liking was increased regardless of whether an increase (left side, Experiment 1) or a decrease (right side, Experiment 2) in the SCR curve indicated a high ease of processing.

A repeated measures ANOVA testing the influence of presentation duration and feedback on liking ratings showed significant main effects of both presentation duration, *F*_(3, 162)_ = 5.86, *p* = 0.001, η^2^_p_ = 0.10, and feedback, *F*_(1.71, 92.11)_ = 5.32, *p* = 0.009, η^2^_p_ = 0.09, but no interaction, *F*_(6, 324)_ = 0.67, *p* = 0.678, η^2^_p_ = 0.01. An analysis of linear trends showed that the longer the stimuli were presented the more they were liked, *F*_(1, 54)_ = 17.14, *p* < 0.001, η^2^_p_ = 0.24 (see Figure 2). For the feedback, a *t*-test showed that stimuli in the high feedback condition (*M* = 3.92, *SD* = 0.96) were liked more than in the low feedback condition (*M* = 3.72, SD = 0.85, *p* = 0.009, see left side in Figure 3). Liking ratings in the no-feedback condition were between high and low feedback (*M* = 3.86, SD = 0.88). However, the mean was only significantly different from low feedback (*p* = 0.011), but not from high feedback. These results show that both the actual ease of processing, manipulated through presentation duration, and the false feedback influenced ratings of liking. Both factors exerted their effects on how much the stimuli were liked. Thus, neither did the feedback overshadow the effects of an actual manipulation of ease of processing nor led the conscious reflection through feedback to discounting of felt fluency as a source for liking.

### Correlations

To also show that evaluations of felt fluency and liking are strongly related, we performed bivariate correlations. For each participant we first correlated the stimulus ratings of liking and felt fluency both over all conditions and separately for all factor combinations. For further analyses the correlations were Fisher *z*-transformed. The mean correlations presented in the following are the retransformed Pearson correlations. A one sample *t*-test showed a significant positive correlation between felt fluency and liking, *r*_(55)_ = 0.42, *p* < 0.001. The correlations computed separately for all factor combinations also show significant positive correlation between felt fluency and liking, *r*s between 0.32 and 0.50, *p*s < 0.001. Furthermore, a 4 (presentation durations of 100, 200, 300, or 400 ms) × 3 (felt fluency feedback high, low, or absent) repeated measures ANOVA with the Fisher *z*-transformed correlations as dependent variables yielded no significant effects. This indicates that the correlations are similar in all conditions.

To sum up, Experiment 1 showed that false feedback influenced the liking ratings of our participants. Supporting the theory of processing fluency, participants awarded higher liking ratings to stimuli in trials where a feedback of high ease of processing was provided than to stimuli in trials where a feedback of low ease of processing was given. In trials where no feedback was given, i.e., where participants could only rely on the actual ease of processing, they awarded liking ratings that fell right between the two feedback conditions. These findings suggest that both feedback and the actual ease contribute to a feeling of fluency that is then used as a source for liking.

However, using an increasing skin conductance curve as a feedback of high ease of processing and a decreasing curve as a feedback of low ease of processing we cannot exclude that the effects on felt fluency and liking were due to compatibility between the direction of the curve and the response (Hommel and Lippa, 1995). In the second experiment, we thus instructed a new set of participants that a feedback of an increasing skin conductance response indicated low ease of processing and a feedback of a decreasing skin conductance response indicated high ease of processing. The no-feedback condition was left unchanged. If the effects were solely due to compatibility then the effects on felt fluency and liking in Experiment 2 should be reversed to Experiment 1.

## Experiment 2: Materials and methods

### Participants

In Experiment 2, 56 undergraduate students from the University of Vienna (45 female, *M*_age_ = 24.9 years, *SD* = 7.8), who participated in return for partial course credit, believed the feedback manipulation and were eligible for further analysis. All participants provided written informed consent prior to participation. None of the participants has participated in Experiment 1.

### Stimuli

The same stimuli as in Experiment 1 were used.

### Design and procedure

In comparison to Experiment 1, in Experiment 2 the meaning of the feedback was reversed. In Experiment 2, a feedback of an increasing skin conductance response represented a *low* ease of processing feedback and a feedback of a decreasing skin conductance response represented a *high* ease of processing feedback. The no-feedback condition remained unchanged. Accordingly, we adapted the introductory texts and the practice trials to reflect the changes in the meaning of the skin conductance response. Everything else was identical to Experiment 1.

## Experiment 2: Results and discussion

To analyze the effects of our manipulations on ratings of felt fluency and liking we performed two separate 4 (presentation durations: 100, 200, 300, or 400 ms) × 3 (felt fluency feedback: high, low, or absent) repeated-measures ANOVAs; once with ratings of felt fluency and once with ratings of liking as the dependent variable. In all analyses, the level of statistical significance was *p* < 0.05. When the sphericity assumption was not met, the degrees of freedom were Greenhouse-Geisser corrected. For presentation duration, linear trend analyses were performed and for feedback, we compared the mean ratings in the low feedback condition with the mean ratings in the high feedback condition employing *t*-tests.

### Felt fluency

**Figure 4:**
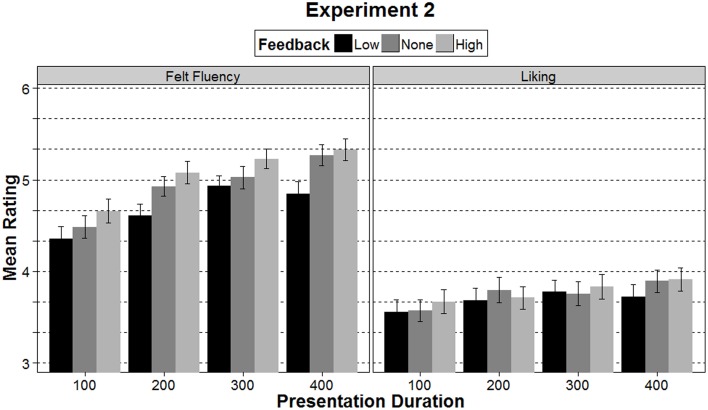
**Mean ratings of felt fluency and liking in Experiment 2 separately for the three feedback conditions and the four presentation durations**. Error bars represent ±1 SE.

An ANOVA with felt fluency as the dependent variable showed significant main effects of both presentation duration, *F*_(2.14, 117.84)_ = 39.52, *p* < 0.001, η^2^_p_ = 0.42, and feedback, *F*_(1.78, 97.62)_ = 22.21, *p* < 0.001, η^2^_p_ = 0.29. The interaction between presentation duration and feedback was not significant, *F*_(6, 330)_ = 1.22, *p* = 0.297, η^2^_p_ = 0.02. Linear trend analysis shows that ratings of felt fluency linearly increased with longer presentation durations, *F*_(1, 55)_ = 57.87, *p* < 0.001, η^2^_p_ = 0.51 (see Figure 4). Thus, the longer the stimuli were presented the higher the subjective feeling of fluency. Also in line with our manipulation, a *t*-test showed that ratings of felt fluency were significantly higher following a high feedback (*M* = 5.12, *SD* = 0.79) than following a low feedback (*M* = 4.72, *SD* = 0.82, *p* < 0.001). The no-feedback condition (*M* = 4.97, SD = 0.77) showed ratings in between the two other conditions (all *p*s < 0.007). These results indicate that (a) an actual higher ease of processing (through variations in presentation duration) was subjectively felt as more fluent and (b) false feedback had the intended effect on felt fluency ratings. This means that although we reversed the mapping, the participants still understood the feedback as intended.

### Liking

Analyzing how the manipulations are reflected in the liking ratings, an ANOVA showed a main effect of presentation duration, *F*_(2.56, 140.70)_ = 9.49, *p* < 0.001, η^2^_p_ = 0.15. Linear trend analysis showed that liking linearly increased with presentation duration, *F*_(1, 55)_ = 18.01, *p* < 0.001, η^2^_p_ = 0.25. For feedback, the analysis revealed a trend, *F*_(1.70,93.41)_ = 2.55, *p* = 0.092, η^2^_p_ = 0.04. This trend is also reflected in a marginally significant difference in a *t*-test between the mean liking ratings in the high (*M* = 3.80, *SD* = 0.89) and low feedback condition (*M* = 3.70, SD = 0.92, *p* = 0.071, see right side of Figure 3). Though not significantly different, the means in the three different feedback conditions indicate that the liking ratings were highest with high feedback, followed by no feedback, and lowest with low feedback (see Table 1 and Figure 4). There was no interaction between presentation duration and feedback, *F*_(4.89, 268.87)_ = 0.91, *p* = 0.471, η^2^_p_ = 0.02.

With a reversed feedback mapping results for liking seem less clear as in Experiment 1. The effects, though more subtle, work in the same direction as in Experiment 1. Our results indicate two things: First, the effects in Experiment 1 are not solely due to compatibility. If this would have been the case then effects in Experiment 2 should have been reversed to those in Experiment 1. Second, compatibility between the direction of the curve (increase) and a higher rating is nonetheless favorable for effects of feedback on liking. The effects were more pronounced in Experiment 1, where both false feedback and compatibility work in the same direction.

### Correlations

The relationship between felt fluency and liking is also reflected in bivariate correlations between ratings of felt fluency and liking for each participant. Overall, felt fluency and liking are significantly related, *r*_(56)_ = 0.46, *p* < 0.001. The correlations computed separately for all factor combinations also show significant positive correlation between felt fluency and liking, *r*s between 0.37 and 0.56, all *p*s < 0.001. Furthermore, a 4 (presentation durations of 100, 200, 300, or 400 ms) × 3 (felt fluency feedback high, low, or absent) repeated-measures ANOVA with the Fisher *z*-transformed correlations as dependent variables yielded a main effect of presentation duration, *F*_(3, 153)_ = 3.52, *p* = 0.017, η^2^_p_ = 0.07. *Post-hoc* pairwise comparisons indicate that the correlations at 100 ms (*r* = 0.54) are significantly higher than at 400 ms (*r* = 0.43, *p* = 0.022). Furthermore, there was a significant main effect of feedback, *F*_(2, 102)_ = 3.48, *p* = 0.035, η^2^_p_ = 0.07. Though the means show that without feedback the correlations tended to be higher (*r* = 0.53) than with high (*r* = 0.46) or low (*r* = 0.46) feedback, in *post-hoc* comparisons none of the differences reached significance (*p*s > 0.081). The interaction between presentation duration and feedback was not significant, *F*_(6, 306)_ = 1.30, *p* = 0.256, η^2^_p_ = 0.03. Though the findings indicate stronger relations at shorter presentation durations and without feedback, the correlations are all significantly different from 0 and in the range of previous findings (Forster et al., 2013).

## General discussion

In two experiments we tested whether differences in subjective feelings of fluency, manipulated through false feedback when viewing stimuli, influence liking of those stimuli. Such a relationship has generally been claimed in the theory of processing fluency (Reber et al., 2004b; Regenberg et al., 2012; Forster et al., 2013), but up to now remained largely untested. Findings in Experiment 1 clearly indicate that the false feedback, in addition to an actual manipulation of ease of processing, contributes to liking evaluations. Our second experiment highlights the boundaries and requirements for the effect. Under incongruent mapping between feedback curve and response direction—when an increase in skin conductance indicated lower ease of processing and thus should lead to lower liking—the effects of the false feedback were weaker, albeit still in the same direction. Thus, the subjective feeling of fluency is used as a source for liking evaluations given the interpretation of the subjective feeling is straightforward for the perceiver. Effects of presentation duration, an actual variation in ease of processing, were consistent across both experiments: the higher the actual ease of processing, the higher the liking.

In Experiment 1 feedback of a high ease of processing increased felt fluency. Thus, participants correctly understood the feedback trials. Furthermore, for ratings of felt fluency a conscious and clear feedback of how easy something was processed did not lead to discounting of this feeling (see also Newell and Shanks, 2007). Our manipulation of ease of processing through variations in presentation duration also produced differences in rating of felt fluency, replicating previous findings (Reber et al., 2004b; Regenberg et al., 2012; Forster et al., 2013). Thus, both manipulations contributed to the effect. Though the higher ease of processing not necessarily needs to be consciously represented (Reber et al., 2002; Topolinski and Strack, 2009), we can nonetheless conclude that both our manipulations were successful—and effective—in eliciting a subjective feeling of fluency.

Regarding our main hypothesis, in both experiments feedback of a high ease of processing increased liking; in the no-feedback condition liking ratings fell in between high and low feedback. This indicates that without feedback participants were affected only by the actual ease of processing based on presentation duration. In the high and low feedback conditions the presentation duration manipulation exerted additive and independent effects, as indicated by the significant main effects of both false feedback and presentation duration, and by the absence of any interactions between the factors. Moreover, false feedback did not override effects of ease of processing through presentation duration. Possibly, the manipulations even activated two different processes: one accessible to conscious reflection (false feedback), another inaccessible to conscious reflection (ease of processing). The results of the felt fluency ratings however cast some doubt on this possibility. If our presentation duration manipulation had been impervious to conscious reflection than those variations should not have been reflected in felt fluency ratings.

In Experiment 2 we tested the preconditions and the stability of the effect by reversing the mapping between the skin conductance response and feedback. An increase in skin conductance now indicated a lower ease of processing. In comparison with Experiment 1, we thus excluded that the effects were due to compatibility between an (increasing) feedback curve and (increasing) liking ratings. For ratings of felt fluency we found nearly identical results showing that the manipulation was successful. Despite the reversal of the feedback curve participants reported higher felt fluency after a feedback of high ease of processing compared to a feedback of low ease of processing. Again the no-feedback condition showed ratings in between the two other feedback conditions.

In Experiment 2, the direction of the effects for liking was according to our hypothesis and in the same direction as in Experiment 1. However, we found slightly weaker effects of high vs. low feedback. This is also indicated by only a marginally significant main effect of false feedback. Similar to Experiment 1, the manipulation of ease of processing through presentation duration showed a main effect. This hints that both factors contribute to liking. We can conclude from Experiment 2 that the compatibility between the direction of the feedback curve and the direction of the rating is a favorable precondition for the effect, but not the sole responsible factor. Thus, compatibility seems to ease the attribution of fluency as the source. From the rating of felt fluency we can conclude that the feedback was correctly interpreted. But the participants did not rely on it as strongly as in Experiment 1 (see also Unkelbach and Greifeneder, 2013). The difference in the number of participants who did not believe the instruction between Experiment 1 (*n* = 8) and Experiment 2 (*n* = 40) also supports the notion that compatibility renders the influence of felt fluency on liking more plausible.

Our correlational analyses in both experiments corroborate the findings just presented. Ratings of felt fluency and liking in both experiments throughout all conditions are significantly related. These results are well in line with previous studies showing that ratings of felt fluency and liking are significantly related (Forster et al., 2013).

As the results of felt fluency and liking in both experiments are in the same direction and as both ratings are provided in each trial, it is hard to clearly disentangle effects of our manipulations on felt fluency from effects on liking. However, in our instructions at the beginning of the experiments we were careful to only stress the relation between feedback of skin conductance and ease-of-processing. Relationships with liking were never mentioned. Furthermore, using the same measures of subjectively felt fluency and liking, Jakesch et al. (2013) could show that subjective fluency and liking dissociate. As both ratings were provided in each trial, it would also be possible that liking influenced the subjective fluency. There are however no theoretical reasons for why evaluations of liking should influence the feeling of fluency. Also Constable et al. (2013) could show that liking evaluations did not influence response fluency. We, thus assume that in accordance with the processing fluency accounts higher ease-of-processing led to higher liking and not vice versa.

Previous studies testing the effects of a subjective feeling of fluency either ensured in pre-studies that the subjective feeling of fluency is sensitive to changes in ease of processing or used ratings of felt fluency as dependent variables (Reber et al., 2004b; Regenberg et al., 2012; Forster et al., 2013; Jakesch et al., 2013). Due to the experimental manipulation with a false feedback eliciting a subjective feeling of fluency we can now more safely conclude that this feeling is used as a possible source for our evaluations.

Of course the ease of processing is but one factor influencing our appreciation of objects (Leder et al., 2004; Bullot and Reber, 2012; Leder and Nadal, 2014). Though not yet empirically shown, other factors such as color (Palmer and Schloss, 2010) or personal taste (Vessel and Rubin, 2010) might well interact with the ease of processing in establishing appreciation. At longer exposure and deeper processing, ambiguous objects that pose challenges in perceiving and understanding are appreciated more than easily perceived and understood counterparts (Jakesch et al., 2013). Nonetheless, first impressions and heuristics—especially, when we have no clear preference or other cues are missing—play a role in our everyday evaluations of the environment (see for example in faces, Bar et al., 2006, or brands, Lee and Labroo, 2004). Employing established procedures of previous research we presented rather simple line drawings. With more elaborate material higher-order cognitive processes such as memory integration, personal taste, or expertise might even override the low-level effects (Leder et al., 2004). How the effects in interact with other factors remains to be shown and is a challenge for further research on the formation of aesthetic appreciation.

**Figure 5:**
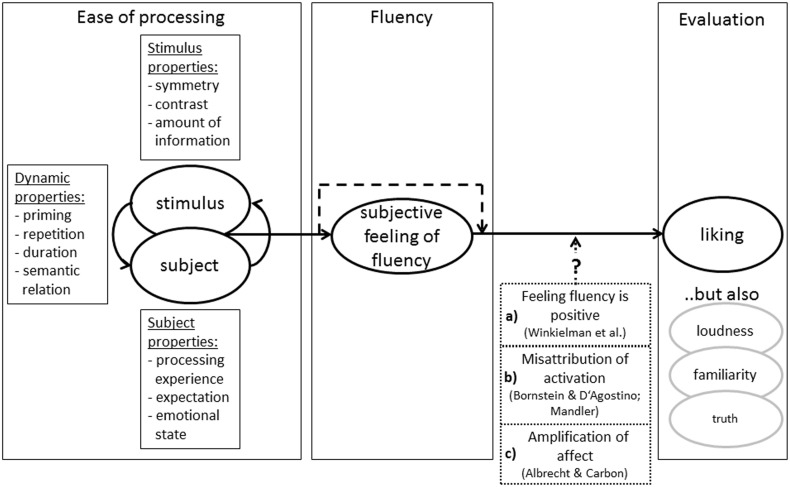
**A schematic model of processing fluency**. Higher ease of processing leads to a subjective feeling of fluency, which in turn influences our evaluations.

Our findings, together with previous research on processing fluency (Reber et al., 1998, 2004a; Winkielman et al., 2003), suggest the following preliminary model of processing fluency (see Figure 5): Interactions between a stimulus and a person looking at a stimulus vary in ease of processing. A number of factors, such as symmetry, priming, or expectations (see Figure 5), contribute to the ease of processing; in our studies variations in presentation duration and false feedback (see also Table 1 in Alter and Oppenheimer, 2009, for a comprehensive overview). Ease of processing leads to a subjective feeling of fluency (Reber et al., 2004b; Regenberg et al., 2012; Forster et al., 2013), which can be consciously reported.

The subjective feeling of fluency is then used as a source of information (see Schwarz, 2011, or Schwarz and Clore, 2007, for an overview). Such a feeling of fluency affects liking, but also other evaluations such as judgments of truth (Reber and Schwarz, 1999) or familiarity (Whittlesea, 1993). In Figure 5 a dashed line from ease of processing to liking indicates that ease of processing can influence judgments also without being consciously perceived (Reber et al., 2002; Topolinski and Strack, 2009).

How the feeling influences our evaluations is not yet fully understood. One approach (Box a in the model) suggests that the feeling is *per se* positive, because it signals a positive state of affairs (e.g., Winkielman et al., 2003). Thus, this feeling leads to more positive ratings. Alternatively, the feeling of fluency is an unspecific activation (Mandler et al., 1987; Jacoby et al., 1988) leading to generally higher evaluations irrespective of the valence of the rating dimension (Box b in the model, Bornstein and D'Agostino, 1992, 1994). Another possibility is that the feeling of fluency amplifies the affective evaluation of the stimuli, but leaves affectively neutral stimuli uninfluenced (Box c in the model, Albrecht and Carbon, 2014). Though most evidence is in favor of the hedonic marking of felt fluency (Fang et al., 2007; Topolinski et al., 2009), conclusive evidence is still lacking (see also Unkelbach and Greifeneder, 2013).

On a theoretical level, the processing fluency account also has its share in the longstanding dissociation between cognition and emotion. In the light of our results, it seems futile to separate such intricately interwoven processes when even such a strictly cognitive process—the perception—has affective components and can influence how much we appreciate our environment (see for example Schwarz and Clore, 2007; Storbeck and Clore, 2007). This tight connection between seeing and feeling is most probably due to a—phylogenetically or ontogenetically—useful preference for the known (Zajonc, 2001; Unkelbach, 2006; Schwarz, 2011). Thus, for aesthetic appreciation we would advise: whenever looking at an artwork, just enjoy the pleasure of the mere perception and indulge in your feeling of fluency.

## Conflict of interest statement

The authors declare that the research was conducted in the absence of any commercial or financial relationships that could be construed as a potential conflict of interest.
