# Supplementary material for: Do I really feel it? The contributions of subjective fluency and compatibility in low-level effects on aesthetic appreciation
Source: Front Hum Neurosci. 2015 Jun 26;9:373. doi: 10.3389/fnhum.2015.00373 (PMC4481155; doi:10.3389/fnhum.2015.00373)
Supplement: Supplementary file 1 [file DataSheet1.DOCX]

***Supplementary Material***

**Do I really feel it? The interplay of fluency and compatibility in low-level effects on aesthetic appreciation**

**Michael Forster^1^*, Wolfgang Fabi ^1^, Helmut Leder ^1^**

^1^Department of Basic Psychological Research and Research Methods, Faculty of Psychology, University of Vienna, Vienna, Austria

*** Correspondence:** Michael Forster, Department of Basic Psychological Research and Research Methods, Faculty of Psychology, University of Vienna, Liebiggasse 5, Vienna, 1010, Austria.

Michael.forster@univie.ac.at

1. **Supplementary Data**

**Analysis of the full sample of participants**

1.1. Experiment 1

1.1.1 Felt fluency

Running the same analysis with the full sample (including the participants who did not believe the feedback manipulation, *n* = 11) showed similar results. Ratings of felt fluency were significantly higher in the high feedback condition (*M* = 5.07, *SD* = 0.76) than in the low feedback condition (*M* = 4.71, *SD* = 0.95, *p* = .001), although the overall effect size for feedback was smaller (η_p_^2^ = .15).

1.1.2 Liking

The same analysis with the full sample (*n* = 69) also showed a main effect for presentation duration and also no interaction, but only a trend for feedback, *F*(1.77, 120.25) = 2.72, *p* = .076, η_p_^2^ = .04. Although inspection of the mean liking ratings shows that in the high feedback condition liking ratings are higher than in the low feedback condition, the absence of a significant effect shows that participants, who did not believe the instruction, did attenuate the effect of the feedback manipulation on liking.

1.2 Experiment 2

1.2.1 Felt fluency

An analysis with the full sample (including the participants, who did not believe the feedback manipulation, *n* = 40) yielded similar findings. Ratings of felt fluency were significantly higher in the high feedback condition (*M* = 4.98, *SD* = 0.82) than in the low feedback condition (*M* = 4.70, *SD* = 0.81, *p* < .001), although the overall effect size for feedback was smaller (η_p_^2^ = .16).

1.2.2 Liking

An analysis with the full sample (*n* = 96) showed similar findings. As in Experiment 1, when including participants who did not believe the feedback manipulation the effects in the feedback condition were attenuated, *F*(2, 190) = 1.56, *p* = .213, η_p_^2^ = .02.
